# The effect of the presence of quiescent female nymphs, males and their spermatophores on spermatophore placement in two species of eriophyoid mites

**DOI:** 10.1007/s10493-013-9657-y

**Published:** 2013-01-18

**Authors:** Katarzyna Michalska, Marcin Studnicki

**Affiliations:** 1 Department of Applied Entomology, Warsaw University of Life Sciences, Nowoursnowska 159, 02-776 Warsaw, Poland; 2 Department of Experimental Design and Bioinformatics, Warsaw University of Life Sciences, Nowoursnowska 159, 02-776 Warsaw, Poland

**Keywords:** Spermatophore, Eriophyoidea, *Aculops allotrichus*, *Aculus fockeui*, Sex dissociation, Male–male competition

## Abstract

Under sex dissociated sperm transfer, females seek spermatophores and pick up sperm without male assistance. In several species males adjust spermatophore deposition rate to the presence of conspecifics. It is not known, however, which factors could favor such elasticity in non-pairing males. In this paper, we compare male response towards conspecifics between the sex dissociated eriophyoid mites *Aculus fockeui* (Nalepa and Trouessart) and *Aculops allotrichus* (Nalepa). The species differ significantly in male reproductive strategies and, consequently, the intensity of male–male-competition. *Aculus fockeui* males deposit spematophores all over the leaves and occasionally leave single spermatophores beside quiescent female nymphs (QFNs). In contrast, *A. allotrichus* males guard QFNs and encircle them with spermatophores. In this study, males of both species deposited spermatophores close to and apart from the rival spermatophores. *Aculops allotrichus* males had similar spermatophore output whether they were kept alone or in a group of seven males. They did not change spermatophore output in the presence of five rival spermatophores, a QFN or a QFN and varying number of rivals, either. In contrast, *A. fockeui* males increased spermatophore output in the presence of rival spermatophores or when on the arena with a QFN the male number increased to eight males. They did not respond, however, to the presence of a QFN and one rival or a QFN alone. The possible effect of the species-specific intensity of male–male competition, population density, the availability of receptive females and the rate of spermatophore output on the flexibility of eriophyoid spermatophore deposition is discussed.

## Introduction

In arthropods that reproduce without pair formation (so-called sex dissociation) males deposit numerous spermatophores on a substrate, while females seek them and accomplish self-insemination. However, spermatophore production can be costly (Dewsbury 1982; Proctor 1998). Thus, non-pairing males can employ various tactics that enable them to increase the rate of contacts between female and spermatophores, and out-compete the rival males. For example, they destroy the rival spermatophores, defend spermatophore fields against intruder males (Proctor 1998; Stam et al. 2002) or guard pre-emergent females and deposit spermatophores close to them (Michalska 1999; Michalska et al. 2010). Moreover, they can prudently allocate their spermatophore expenditures, increasing or decreasing their spermatophore output according to the presence of females (e.g. Witte 1991; Proctor 1992; Michalska et al. 2010), males (e.g. Witte 1991; Proctor 1992; Michalska 2000) and already deposited spermatophores (Proctor 1998). It is not known, however, what factors could favor the evolution of such elasticity of spermatophore deposition in non-pairing males.

Eriophyoid mites are gregarious herbivores that form galls or live freely on plants and can cause serious losses in the yield and quality of many crop plants (Lindquist et al. 1996). Similarly to some other prostigmatic or oribatid mites, they are involved in dissociated sperm transfer (Thomas and Zeh 1984; Oldfield and Michalska 1996; Proctor 1998; Michalska et al. 2010).

In several eriophyoid species, spermatophores have been found in groups (Michalska et al. 2010). Such aggregative distribution of sperm, also recorded in other arthropods with dissociated sperm transfer, may be regarded as a consequence of competition between males. However, by depositing spermatophores close to the previously deposited, usually older, spermatophores, males can also steal the pheromonal output of rival spermatophores and increase the attractiveness of their own spermatophores for the seeking females (Proctor 1998).

The characteristic feature of some eriophyoid species is male interest in pre-emergent females, so-called quiescent female nymphs (QFNs), and the deposition of spermatophores close to them (Michalska and Boczek 1991; Michalska et al. 2010). It seems to be connected with single or very rare inseminations in a female lifetime. In *Aculus fockeui* (Nalepa and Trouessart) and *Aculops allotrichus* (Nalepa) (synonym *Vasates robiniae*), in which males attend quiescent female nymphs, *A. fockeui* females picked up sperm from only a single spermatophore during their lifetime, while nearly 30 % of *A. allotrichus* females performed self-insemination repeatedly and visited two spermatophores in their lives (Michalska and Mańkowski 2006). In both species, the males can also deposit spermatophores in isolation, while the emergent females pick up sperm from spermatophores without male assistance.

The previous study revealed some elasticity in the spermatophore deposition of eriophyoid males. Young, 2–3 day old *A. fockeui* males that had been reared from the quiescent nymph stage in isolation from conspecifics, deposited significantly less spermatophores when they were kept in groups of seven males than those isolated from other males (Michalska 2000). By contrast, males that were randomly collected from a population, varied in age and had experienced contacts with other males during their adulthood did not show such a response (Michalska 2005). Also in *Cecidophyopsis hendersoni* (Keifer), neither the presence of males nor spermatophores affects spermatophore production in randomly collected males. However, the presence of virgin females incited a higher rate of spermatophore deposition in these males (Michalska and Shi 2004).

In this paper we examine the effect of the presence of quiescent female nymphs, males and their spermatophores on spermatophore deposition rate in randomly collected males of two species of eriophyoid mites: the above-mentioned *A. fockeui* from peach and *A. allotrichus* from black locust trees. We also inspect how males of these eriophyoids distribute their spermatophores on leaf arenas where spermatophores have previously been deposited by rivals; whether males preferentially deposit spermatophores into groups with rival sperm and/or form groups of their own spermatophores.

Both species form similarly dense populations on leaves (Michalska pers. obs.). However, they differ considerably in male reproductive strategies, and the intensity of male–male competition. In *A. fockeui,* males visit quiescent female nymphs, for one or two minutes only, and occasionally deposit single spermatophores beside them (Oldfield and Michalska, 1996). By contrast, in a ‘guarding’ species, *A. allotrichus*, males encircle quiescent female nymphs with spematophores and can guard them for many hours, solitary or jointly (up to several males beside a nymph), until female emergence (Michalska, 1999). In *A. fockeui* the proportion of males among the adult population lies between 0.2 and 0.3 (Putman 1939), while in *A. allotrichus* it reaches on average 0.6 (Michalska and Mańkowski 2006). Thus, the male supply in a population and, consequently, the intensity of competition between males, appears to be much higher in *A. allotrichus* than in *A. fockeui*. We hypothesize that the different, species-specific, intensity of male–male competition may differently shape male response to the presence of conspecifics as well as flexibility in the spermatophore production and spermatophore distribution in these mites.

## Materials and methods

### Mites, rearing and experimental setup

*Aculus fockeui* occurs freely on leaves of various species belonging to the genus *Prunus*. It is an important pest of plum and peach (Castagnoli and Oldfield 1996). *Aculops allotrichus* inhabits leaves of the black locust tree *Robinia pseudoacacia* L. (Michalska 1999). To the knowledge of the authors, the phylogenetic relationship between *A. fockeui* and *A. allotrichus* has not been investigated.

In this study, the stock population of *A. fockeui* came from *Prunus persica* cv. ‘Iskra’ grown in Warsaw Botanical Garden, Powsin and *A. allotrichus,* from *R. pseudoacacia* grown on the campus of Warsaw University of Life Sciences. Only *A. fockeui* was mass-reared. In the case of *A. allotrichus*, there were difficulties in maintaining the detached leaves of a host-plant under laboratory conditions for a period of several days, which precluded the mass-rearing of this eriophyoid (see also de Lillo et al. 2010). *Aculus fockeui* was reared in detached-leaf cages (a rearing chamber of 2 cm in diam) as described by Oldfield et al. (1970), on the underside of leaves of a ‘Rakoniewicka’ peach, grown in the field of Warsaw University of Life Sciences. For experiments we used one-chamber cages (leaf arena of 0.55 cm in diam), whereas to rear quiescent female nymphs or temporarily isolate males before test ‘rearing’, four-chambered cages (each leaf arena of 0.65 cm in diam) were applied (for details see Michalska 2005). For all cages, we used fresh and ‘clean’ leaves. They were collected from trees that were not infested by eriophyoids. Cages with mites were maintained in a Sanyo MLR Plant Growth Chamber, at 26 °C, 85–90 % RH, and 16/8 L/D photoperiod. Mites and spermatophores were manipulated using a human eyelash glued to a wooden stick under a stereo microscope (at 50–100× magnification) aided by a cold light source.

For the tests, males of both species were collected randomly from leaves and then kept in solitude on leaf arenas for 24 h. Using this procedure, the conditions experienced by the males before experiments were unified. Moreover, it enabled us to diminish the variation of spermatophore production by *A. allotrichus*. Only on the second day of isolation from conspecifics did the coefficient of variance of spermatophore deposition rate of *A. allotrichus* decrease significantly, and it did not differ from that of *A. fockeui* (Michalska 2012).

Males were primarily distinguished from females on the basis of sexual dimorphism. In *A. fockeui* and *A. allotrichus,* males are slender and slightly shorter than females. For tests we chose only those eriophyoids, which deposited at least one spermatophore during the 24-h period of solitude.

As previous studies have revealed (Michalska 2011), males of *A. allotrichus* have a low rate of spermatophore deposition, much lower than that of *A. fockeui*. In isolation from conspecifics, *A. fockeui* males deposited on average 19.1 spermatophores per day while *A. allotrichus* deposited 3.6 spermatophores only. Thus, to obtain a larger number of spermatophores of *A. allotrichus* (e.g. in the experiments in which the pattern of spermatophore distribution was examined) tests were prolonged to 10 h. Only in the experiments with quiescent female nymphs, similarly as in the case of *A. fockeui*, tests with *A. allotrichus* lasted 5 h.

The previous observations on joint-guarding of QFNs by *A. allotrichus* males have shown that males of this species become especially attracted to QFNs and increase their activity a few hours prior to female emergence (Michalska et al. 2010). In *A. fockeui*, however, the attractiveness of QFNs has not so far been investigated. We assumed that, similarly as in some insect and mite species (see, for review Michalska 1999), eriophyoid males might be more attracted by ‘older’ pre-emergent females. Thus, in this study, for both species we used ‘old’ female quiescent nymphs, a few hours before moulting. To prepare them for tests, pre-quiescent female nymphs (ca. 1–2 h before quiescence, markedly bigger than male nymphs, convex and shiny, firmly attached with an anal sucker to the leaf) were placed in groups of several individuals on leaf arenas in the afternoon of the day prior to the tests. In both species, the quiescent period of female nymphs lasts only a few hours at 26 °C (e.g. the mean ± SE time of the quiescence of *A. allotrichus* female nymphs at 26 °C and 80–85 % RH was 774.6 ± 6.71 min; N = 10, Michalska pers. obs.). Prior to the tests, female nymphs were maintained at 19 °C to prolong their quiescence and preclude the emergence of females before the tests were completed. Just before the experiments started they were transferred to the experimental cages and re-attached to the leaf with their anal sucker in the center of the leaf arena.

In *A. allotrichus,* the pattern of spermatophore placement throughout the day has not so far been investigated. In contrast, the tests on *A. fockeui* revealed that males of this species deposit many more spematophores in the morning than in the afternoon (Michalska 2005). To avoid the possible effect of time of day on spermatophore deposition rate in this study, males of both species were tested at the same time of day (in the morning). The experiments started at 8–9 a.m. Males were allowed to deposit spermatophores under light conditions. After that time, males were removed from their cages and their spermatophores were counted.

### Impact of the presence of a QFN on spermatophore deposition rate

To estimate the effect of the presence of a QFN on spermatophore deposition rate, single males of *A. allotrichus* and *A. fockeui* were tested in two situations: (1) with and (2) without a single QFN on a leaf arena.

### Effect of the presence of rival males on spermatophore deposition rate

The impact of the presence of rival males on spermatophore deposition by *A. allotrichus* was estimated by comparing the spermatophore output of males that were kept in solitude and in the groups of seven males. For both treatments, the sample size (7 × 1 male per cage and 1 × 7 males per cage) was seven males. Each combination was repeated k = 5 times. The means of mean numbers of spermatophores per male and for each combination were compared.

### Effect of a QFN and varying number of rival males on spermatophore output

To estimate the effect of both the presence of QFNs and rivals on spermatophore deposition rate, males of both species were tested in the following combinations: (1) one male (2) two males and (3) eight males on a leaf arena with a single QFN. As it was not possible to carry out the entire test within 1 day, the test was blocked to minimize variation error (Grafen and Hails 2003). Each block (replication) was 1 day of the test. *Aculus fockeui* males were tested in eight blocks and *A. allotrichus* males, in ten blocks. On each day, the sample size of males per treatment combination was n = 8; the ‘one male’ combination was replicated 8 times (8 × 1 male per cage), the ‘two males’ combination was repeated 4 times (4 × 2 males per cage) and the ‘eight males’ combination was performed once (1 × 8 males per cage). We compared the total spermatophore output of all the males, in each treatment combination.

### Effect of the presence of spermatophores on spermatophore deposition rate

The impact of the presence of spermatophores on spermatophore placement was investigated by comparing spermatophore output of single males in two combinations: on a ‘clean’ leaf arena (control) and on the arena with five spermatophores deposited by other males. Due to the relatively low rate of spermatophore deposition by *A. allotrichus,* the experimental cages for this species were prepared in the evening of the day preceding the test. Single males were released into half of these cages, and allowed to deposit spermatophores. The remaining cages were used for the control combination. All cages were kept for several hours at 26 °C. Prior to experiments, males and spermatophores, except for 5 randomly chosen spermatophores on each leaf arena, were removed. Experimental males were then released into the prepared cages. For *A. fockeui*, the procedure was similar, except that the arenas were prepared in the early morning and males deposited spermatophores for a few hours until the tests started.

### Spermatophore distribution on the leaf arena with rival spermatophores

Both the five spermatophores previously deposited by other males and those deposited by the experimental males, were additionally depicted by drawings. A spermatophore was regarded as a ‘grouped spermatophore’ if it was deposited by the experimental male close to his own or a ‘rival’ spermatophore, at a distance of approximately one or less than one length of a male body from that spermatophore. Otherwise, it was treated as deposited apart from the other spermatophores (a ‘solitary’ one). Similarly we regarded spermatophores as those deposited close to the ‘rival’ spermatophores or apart from them (but deposited close to males’ own spermatophores, or left in solitude). On rare occasions, the location of spermatophores was ‘unclear’ (e. g. they were very close to the assigned border), and these spermatophores were discarded from the analysis. We also examined whether the rival spermatophores were destroyed by the sojourning male e.g. trampled, crushed or broken by the sojourning male.

### Statistical methods

The statistical analyses were carried out using SAS 9.3 (SAS Institute 2012). The data on the impact of the presence of a QFN, males or spermatophores on spermatophore production were analyzed by applying a generalized linear model (GLM) with the Poisson distribution (PROC GENMOD). The Wald test was used to determine whether the effects were significant (Littell et al. 2006).

We tested the impact of a QFN and a varying number of rivals on spermatophore output using a generalized linear mixed model (GLMM) with the Poisson distribution (Littell et al. 2006). The PROC GLMMIX was used for this analysis, where block was treated as a random effect and treatment as a fixed effect. To find out which means were significantly different from one another, we used the CONTRAST options (SAS Institute 2012).

The distribution of ‘solitary’ and ‘grouped’ spermatophores was analyzed using the χ^2^ test of goodness of fit. We analyzed the distribution of spermatophores deposited by N = 23 males of *A. fockeui* (5-h test) and N = 16 males of *A. allotrichus* (10-h test). As samples were homogeneous in both species (heterogeneity χ^2^ test; *A. fockeui*: χ^2^ = 27.925, *df* = 22, *P* = 0.19; *A. allotrichus*: χ^2^ = 19.19, *df* = 15, *P* = 0.21), the χ^2^ test on pooled data was carried out (Zar 1996). The expected proportion of ‘solitary’ and ‘grouped’ spermatophores was 0.5. As the number of degrees of freedom was *df* = 1, the Yates correction for continuity was applied (Zar 1996).

The data were shown as adjusted means and 95 % confidential intervals estimated from a binomial distribution.

## Results

**Fig. 1:**
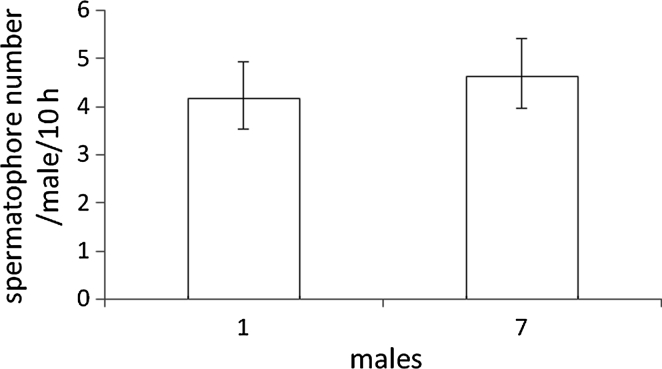
Spermatophore deposition rate of a male of *Aculops allotrichus* kept in solitude or in a group of seven males. (k = 5 replications)

**Fig. 2:**
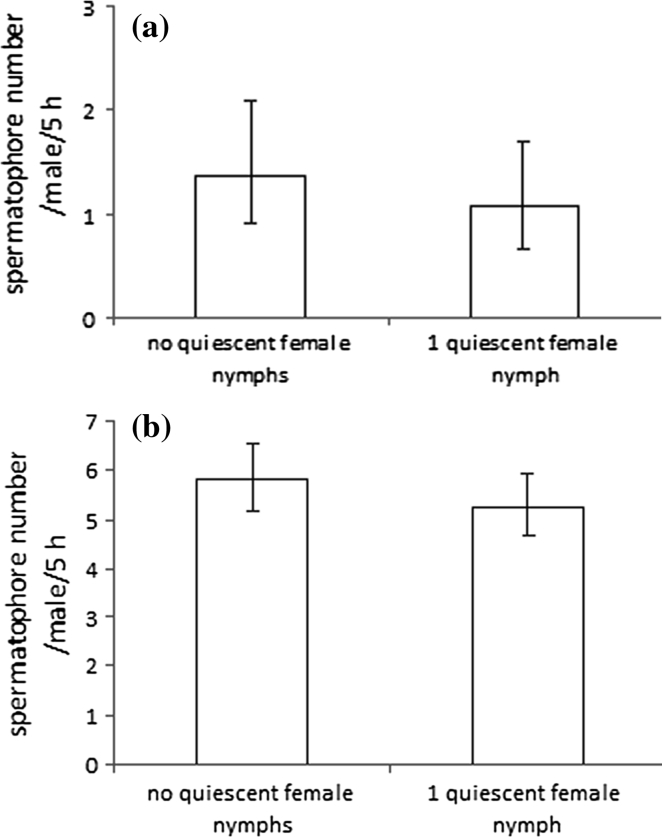
Impact of the presence of a single female quiescent nymph on spermatophore deposition rate of a male of **a** *Aculops allotrichus* (N = 16) and **b** *Aculus fockeui* (N = 49*)*

*Aculops allotrichus* males deposited a similar number of spermatophores whether they were maintained in groups of seven males or in isolation from other males (GLM: deviance = 0.83, *P* = 0.36) (Fig. 1). The number of spermatophores deposited by single males in the presence of a QFN and without a QFN did not differ significantly in either *A. allotrichus* (GLM: deviance = 0.64, *P* = 0.43) (Fig. 2a) or *A. fockeui* (GLM: deviance = 1.34, *P* = 0.25) (Fig. 2b).

**Fig. 3:**
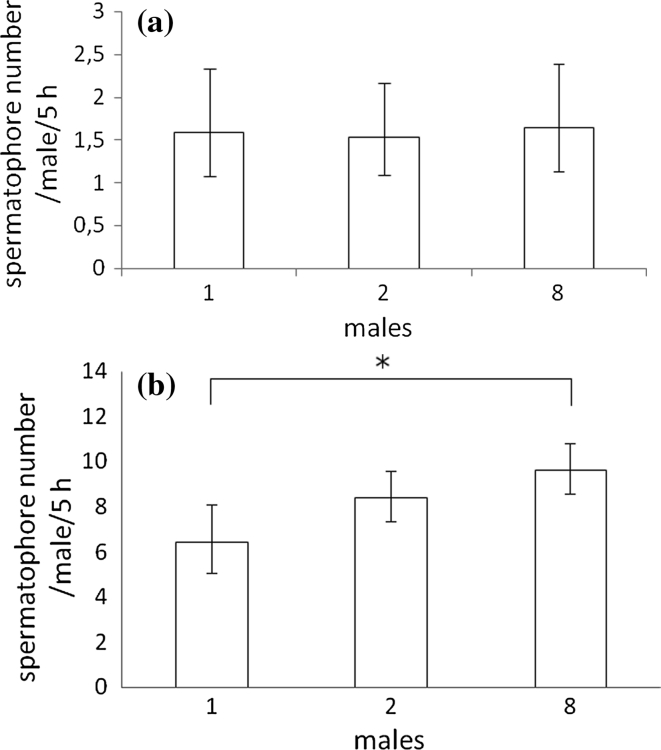
The mean ± C.I. of the mean number of spermatophores deposited by a male of **a** *Aculops allotrichus* (k = 10 replications) and **b** *Aculus fockeui* (k = 8 replications) in the presence of a quiescent female nymph in the following situations: a male in solitude, and a male in a group of either two or eight males. *P ≤ 0.05

In the presence of a QFN and a varying number of rival males, both species differ in allocation of spermatophores. In *A. allotrichus*, there was no significant effect of the presence of rivals on the number of spermatophores deposited by males (GLMM: deviance = 0.16, *P* = 0.85) (Fig. 3a). By contrast, the presence of rivals significantly influenced the spermatophore expenditure of *A. fockeui* males (GLMM: deviance = 19.74, *P* < 0.0001) (Fig. 3b). A pairwise comparison of the means revealed that *A. fockeui* males held in a group of eight males deposited significantly more spermatophores than those without competitors (*P* = 0.027). In the group of two males, the spermatophore output of a male did not differ significantly from that of males held without rivals (*P* = 0.14). There were no significant differences in the number of spermatophores deposited by males in the groups of two and eight males either (*P* = 0.11).

**Fig. 4:**
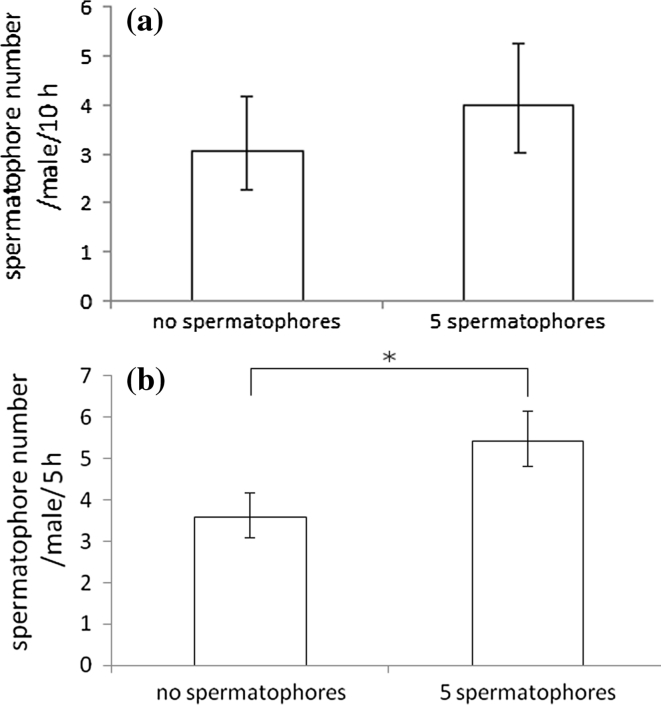
Impact of the presence of spermatophores on spermatophore deposition rate of a male of **a** *Aculops allotrichus* (N = 13) and **b** *Aculus fockeui* (N = 49; *P ≤ 0.05)

The single males of *A. fockeui* placed significantly more spermatophores on the arenas with previously deposited spermatophores than on the ‘clean’ leaves (GLM: deviance = 18.07, *P* < 0.001)) (Fig. 4b). In contrast, the presence of spermatophores did not affect the spermatophore deposition rate of *A. allotrichus* males (GLM: deviance = 1.56, *P* = 0.21) (Fig. 4a).

Males, of either species, did not destroy spermatophores previously deposited by other males. In the test with five spermatophores of other males on the leaf arena, they placed similar numbers of spermatophores close to the ‘rival’ spermatophores and apart from them (Table 1). Beside “mixed” groups of spermatophores, they also formed groups of their own sperm, often at quite long distances of several male body lengths from previously deposited spermatophores. In both species, spermatophores were significantly more often ‘grouped’ (together with male’s own or rival spermatophores) than ‘solitary’ (*A. allotrichus*: χ_adj_^2^ = 36.89, *P* < 0.0001 and *A. fockeui:* χ_adj_^2^ = 50.246, *P* < 0.0001) (Table 1).

**Table 1:** Spermatophore distribution by males of *Aculops allotrichus* and *Aculus fockeui* on the leaf arena with five spermatophores previously deposited by rival males

Species	Number of males	Total numer of spermatophores			
		Grouped versus solitary		Close to versus apart from rival spermatophores	
*A. allotrichus*	16	69	13	44	38
*A. fockeui*	23	90	28	59	59

## Discussion

In this study, males, of either species, did not change spermatophore deposition rate when they were exposed to the presence of quiescent female nymphs. However, the males of each species differ significantly in their response to the presence of rival males and their spermatophores. In contrast to *A. allotrichus*, in which males did not change spermatophore output, whether in the presence of rivals, their spermatophores, or a female quiescent nymph and rivals, *A. fockeui* males increased spermatophore deposition rate in the presence of previously deposited spermatophores or the simultaneous presence of a quiescent female nymph and rival males.

As previous observations showed, males of *A. allotrichus* and *A. fockeui* tend to deposit spermatophores close to quiescent female nymphs (Michalska and Boczek 1991; Michalska 1999; Michalska et al. 2010). Undoubtedly, it can pay for non-pairing males to place spermatophores near receptive or pre-emergent females when the probability that a female picks up sperm from the spermatophore is low, i.e. when there are many competing males that deposit spermatophores within a patch and/or the availability of receptive females within the patch is not constant or predictable. In some water mites, males did not deposit spermatophores in isolation from receptive females and their odours (Proctor 1998). In others, such as in the eriophyoid mite *C. hendersoni*, males increased the rate of spermatophore deposition when receptive females were present (Michalska and Shi 2004). In this species, females are polyandrous and visit several spermatophores in their lifetime (Michalska pers. obs., after Michalska and Mańkowski 2006). Acceleration of the rate of spermatophore deposition is then necessary for males to respond immediately after they encounter a receptive female. In contrast, in *A. fockeui* and *A. allotrichus*, the quiescent period of female nymphs to which males are attracted lasts, according to the temperature, several hours or more (Michalska and Boczek 1991; Michalska pers. obs.) Thus, males do not need to deposit spermatophores immediately after they encounter quiescent female nymphs, as there is still a high probability that the nymphs will be also present some hours later and the spermatophore deposited within this period will also be readily visited by newly emergent females.

In some water mites, e.g. *Hydrachna conjecta* Koenike, *H. magnisculata* Marshall, *Limnochares aquatica* (L.) or *L. americana* Lundblad the presence of other males alone stimulated males for spermatophore deposition (Proctor 1992). However, both, *A. allotrichus* (in this study) and *A. fockeui* (in the previous study, Michalska 2005) behaved differently and did not respond to the presence of competitor males. It is likely that for eriophyoids, in general, due to their relatively low rate of spermatophore deposition (e.g. *A. fockeui* ca. 1–2 per hour and *A. allotrichus*, ca. 0.2–0.8 per hour, in this study), the presence of males alone is not yet a reliable cue of the risk of spermatophore competition. The encountered males may not deposit a spermatophore within a patch, and go away. In *A. fockeui*, only the simultaneous presence of both males and quiescent female nymphs, close to which males tend deposit spermatophores, seems to considerably increase this chance. As this study shows, however, the presence of a single competitor in the vicinity of *A. fockeui* male was still not sufficient for such stimulation. Only the presence of a quiescent female nymph and several males incited a higher rate of spermatophore deposition. Under such circumstances, the probability that any competitor male in a group will deposit a spermatophore at any given time is also considerably increased.

It must be stressed that non-pairing males can also decrease the rate of spermatophore deposition in the presence of other males. In high male densities, males of the oribatid mite *Pergalumna* sp. deposited less spermatophores than at lower male densities (Oppedisano et al. 1995). In the water mite, *Limnesia maculata* (Müller), grouped males kept at lower temperatures had a higher spermatophore output than single males, Rutkis (1987, after Witte 1991). However, at higher temperatures, grouped males markedly impeded spermatophore rate in comparison with single males. Also, young 2–3 day old males of *A. fockeui* when kept in a group of seven males decreased the rate of spermatophore deposition (Michalska 2000). Perhaps, when the rate of contacts between males is high within a patch (e.g. at high densities, higher temperatures or when males are young and vigorous such as in case of *A. fockeui*) the risk of spermatophore competition may be so high that deposition of spermatophores may not pay for males, and this forces them to abandon a patch. Also, the increased activity of grouped males and frequent interactions between them might lead to the decrease of energy reserves that are required for spermatophore production, which could also result in a decrease in spermatophore deposition rate.

In several species of non-pairing mites and pseudoscorpions, instead of destroying spermatophores, males begin to deposit spermatophores after they encounter a spermatophore (Proctor 1998). This can result in the formation of an aggregation of spermatophores that came from various males and are of different ages. As this study revealed, males of both eriophyoid species formed aggregations of spermatophores. Without any preference, they added their spermatophores to spermatophores deposited by other males, or formed an aggregation of their own sperm. Only *A. fockeui* males accelerated spermatophore deposition rate after they encountered previously deposited spermatophores.

The pheromonal plum emitted by a spermatophore aggregation may be larger and more easily detected by a female than that of a single spermatophore. Thus, by inserting a spermatophore into a group of spermatophores, a male can increase the chances that a female find his spermatophore (Proctor 1998). Moreover, the newly deposited spermatophore is probably one of the few fresh spermatophores within a group and, therefore, highly attractive for a female. However, to compete successfully and outnumber the spermatophores of competitors, eriophyoids should accelerate spermatophore output, add as many spermatophores as possible to each encountered spermatophore group and also spread them widely all over the leaf for a chance of attracting receptive females, they also need to deposit sperm in places where there are no spermatophores at all. In *A. allotrichus*, the lack of acceleration of spermatophore output in the presence of previously deposited spermatophores, males, or males with quiescent female nymphs, suggests that in this species guarding quiescent female nymphs and hindering other males from the placement of spermatophores near the nymphs may be more a profitable tactic than competing with other males over the number of deposited spermatophores. It must be also stressed that in contrast to *A. fockeui*, *A. allotrichus* males have a relatively low spermatophore deposition rate (probably as a consequence of secondary association with the pre-emergent females) (Michalska 2011), which may preclude a quick response to males and their spermatophores. Finally, the flexibility of spermatophore deposition may be unprofitable for *A. allotrichus* males, due to the relatively constant presence of a high number of competitors in a population. In both *A. allotrichus* and *A. fockeui*, density of population increases rapidly during the season and can be very high in the summer and autumn months (Michalska pers. obs.). However, in *A. fockeui,* population sex ratios are skewed toward females, while in *A. allotrichus,* female-skewed sex ratios can be observed only in May, at the beginning of the mite development on black locust trees. In the later months, *A. allotrichus* males usually significantly outnumber females and dozens of them can be found on leaves (Michalska and Mańkowski 2006; Michalska pers. obs.). For comparison, in *C. hendersoni,* in which males did not change spermatophore output in the presence of spermatophores, or males either, the male proportion in a population was lower than in *A. allotrichus* and reached on average 0.4 (Michalska and Shi 2004; Michalska and Mańkowski 2006). Nonetheless, this species usually forms very dense populations on yucca leaves (Michalska and Shi 2004), much denser than those of *A. fockeui* or *A. allotrichus*. Under such circumstances, again, there is a constantly high number of rivals in the eriophyoid population and the flexibility of spermatophore deposition in relation to competitor males and their spermatophores may be selected against.

In *A. allotrichus*, males mostly placed spermatophores into groups around female quiescent nymphs (Michalska 1999). However, as this study reveals, they can also form spermatophore aggregations when the nymphs are temporarily absent from a leaf. As some *A. allotrichus* females in a population pick up sperm from two spermatophores in their lifetime (Michalska and Mańkowski 2006), the grouped spermatophores might be readily visited by such repeatedly receptive females.

Summarizing, this study showed that eriophyoid males can respond to the presence of competitors and increase the spermatophore deposition rate when they are exposed to the presence of previously deposited spermatophores or a quiescent female nymph and other males. It depends, however, on the species. In the future, more comparative studies are needed to determine whether such species-specific factors as density of population, availability of receptive females, intensity of male–male competition or the rate of spermatophore output may influence the evolution of flexibility in spermatophore deposition of eriophyoid males. Also, more attention should be paid to the number of male–male contacts and the locomotory activity of males at various male densities, and how this could affect their spermatophore deposition rate.
